# Next generation calmodulin affinity purification: Clickable calmodulin facilitates improved protein purification

**DOI:** 10.1371/journal.pone.0197120

**Published:** 2018-06-04

**Authors:** Julia G. Fraseur, Tamara L. Kinzer-Ursem

**Affiliations:** Weldon School of Biomedical Engineering, Purdue University, West Lafayette, Indiana, United States of America; University of California, Davis, UNITED STATES

## Abstract

As the proteomics field continues to expand, scientists are looking to integrate cross-disciplinary tools for studying protein structure, function, and interactions. Protein purification remains a key tool for many characterization studies. Calmodulin (CaM) is a calcium-binding messenger protein with over a hundred downstream binding partners, and is involved in a host of physiological processes, from learning and memory to immune and cardiac function. To facilitate biophysical studies of calmodulin, researchers have designed a site-specific labeling process for use in bioconjugation applications while maintaining high levels of protein activity. Here, we present a platform for selective conjugation of calmodulin directly from clarified cell lysates under bioorthogonal reaction conditions. Using a chemoenzymatically modified calmodulin, we employ popular click chemistry reactions for the conjugation of calmodulin to Sepharose resin, thereby streamlining a previously multi-step purification and conjugation process. We show that this “next-generation” calmodulin-Sepharose resin is not only easy to produce, but is also able to purify more calmodulin-binding proteins per volume of resin than traditional calmodulin-Sepharose resins. We expect these methods to be translatable to other proteins of interest and to other conjugation applications such as surface-based assays for the characterization of protein-protein interaction dynamics.

## Introduction

Calmodulin is a highly conserved calcium (Ca^2+^) binding protein that plays a role in sensing the frequency and duration of Ca^2+^ second messenger signals responsible for intracellular and intercellular communication, as well as activating and facilitating various protein-protein interactions [1]. Upon binding of Ca^2+^, CaM undergoes a conformational shift, which opens up several hydrophobic patches that facilitate binding to many downstream proteins (over 100 have been identified [2–4]). This binding is readily reversed by chelation of the Ca^2+^ with EGTA. CaM mediates numerous calcium signaling process in the body, and many Ca^2+^/CaM-activated proteins have been studied for decades, yet there remain a number whose function and physiological roles have yet to be fully characterized [3, 5].

CaM affinity purification is widely used to purify many calmodulin (CaM) binding proteins and proteins that are engineered to carry a calmodulin binding peptide [6–8]. Tandem affinity purification protocols also employ a CaM affinity purification step [9]. CaM affinity resins can be readily made by conjugating purified CaM to cyanogen bromide (CNBr) activated resins [10] and are commercially available; a well-known example is Calmodulin Sepharose 4B Fast Flow (GE Healthcare Life Sciences). As these CaM resins are either time consuming to produce or are purchased at a premium, we sought to develop a next generation CaM affinity resin that is readily produced and performs as well as or better than traditional CaM affinity resins.

We have developed an engineered CaM protein that carries a covalently attached azide-moiety (12-ADA CaM) that is active at wild-type levels [4, 11]. The azide moiety allows for selective conjugation of 12-ADA CaM to alkyne-functionalized surfaces with a well know click chemistry reaction, azide-alkyne cycloaddition [4]. In this work, we report the optimization and validation of a novel CaM affinity resin that can be used to isolate target proteins from complex mixtures in a one-step process. We take advantage of 12-ADA CaM to develop a CaM affinity resin that is produced without purification of 12-ADA CaM prior to covalent attachment to the resin. Furthermore, we show that this second-generation CaM affinity resin is highly selective in the purification of CaM-binding proteins, is stable over multiple uses, and is able to purify more protein per mL resin than commercially available CaM affinity purification resin. The general workflow used to generate this resin could be applied to readily produce other protein-conjugated resins that can be used in a number of downstream purification applications.

## Materials and methods

### Synthesis of 12-Azidododecanoic Acid (12-ADA)

**Fig 1:**
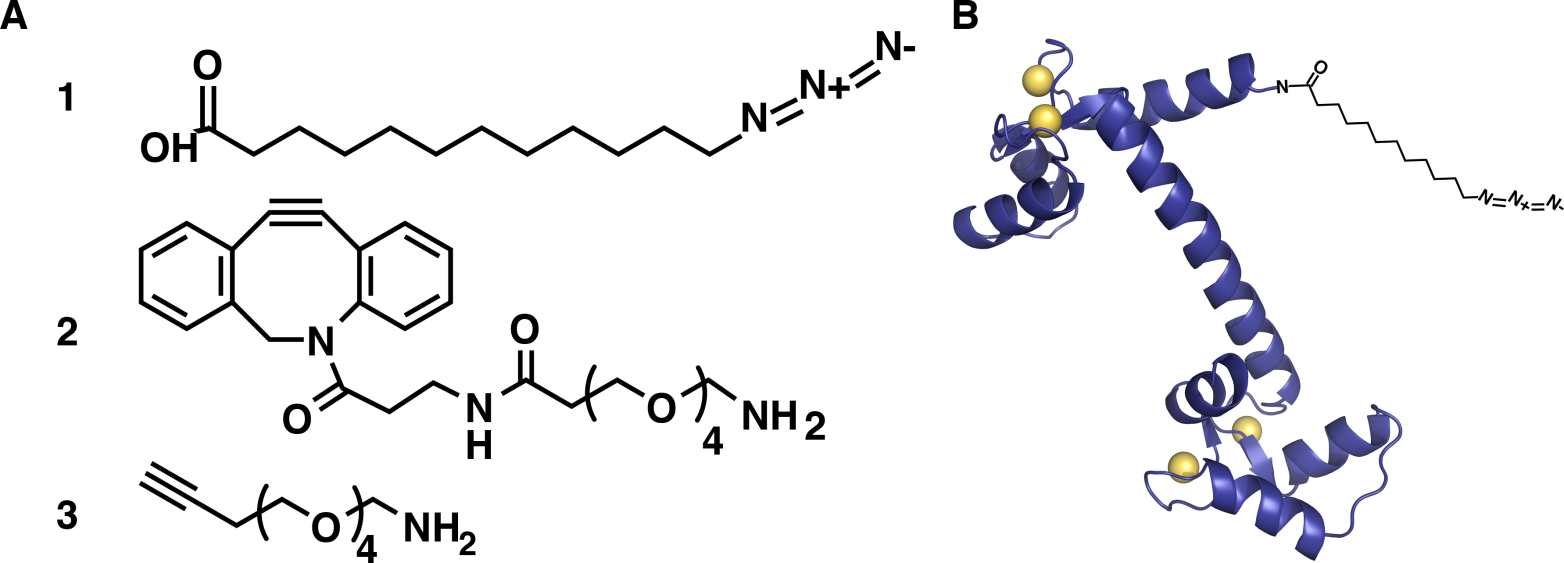
Reagents used in this study. **(A)** Click chemistry reagents used in this study. **1.** 12-azidododecanoic acid (12-ADA), **2.** dibenzocyclooctyne-(polyethylene glycol)_4_-amine (DBCO-PEG_4_-amine), **3.** alkyne-(polyethylene glycol)_4_-amine (alkyne-PEG_4_-amine). **(B)** calmodulin rendered as a ribbon structure with 12-ADA covalently attached to the amino-terminus (not to scale). PDB ID = 1CLL [14].

12-ADA (Fig 1A) was synthesized according methods by Devadas *et al*. and Kulkarni *et al*. with minor modifications [4, 12]. Briefly, 12-bromododecanoic acid (1.8 g, 0.0064 mol), sodium azide (1.2 g, 0.019 mol) and 18-crown-6 (0.5 g, 0.0019 mol) were stirred in 25 mL N,N-dimethylformamide (DMF) under argon at room temperature overnight. DMF was removed under vacuum. The residue was washed with 25 mL dichloromethane, followed by the addition of hydrochloric acid (1 M, 25 mL) to quench unreacted sodium azide. The organic layer was rinsed three times with 25 mL water, dried with Na_2_SO_4_, filtered and concentrated under vacuum. The desired product was a pale-yellow solid at 95% purity (1.5 g, 97% yield). Characterization of the final product (^1^H NMR) matched published results [13] ^1^H NMR 3.19 (t. 2 H, J = 6.9 Hz, CH_2_); 2.28 (t. 2H, J = 7.5 Hz. CH_2_): 1.53 (m. 4H, 2 x CH_2_); 1.21 (m. 14 H) plus 18-crown-6 as a residual impurity ^1^H NMR 3.51(s. 2 H, CH_2_).

### Expression of CaM and Ca^2+^/CaM binding proteins

Cloning, protein expression and tagging were executed according to previously published methods [4]. 12-ADA CaM is a CaM from *Drosophila melanogaster* in a pET-15b plasmid that was engineered to carry a N-myristoyl transferase recognition peptide at the N-terminus as previously described [4] (schematically represented in Fig 1B). Human calcineurin (CaN) in a pET-15b plasmid was obtained from Addgene (reference number 11787) [15]. Human calcium/calmodulin-dependent kinase II alpha (CaMKII) was engineered for *E*. *coli* expression and provided in a pD444-SR plasmid by DNA2.0 (now ATUM, Newark, CA). Plasmids were transformed into chemically competent BL21(DE3) *E*. *coli* and expression cultures were grown in an incubator (250 rpm) at 37°C in LB media supplemented with 50 μg mL^-1^ kanamycin and 100 μg mL^-1^ ampicillin (CaM and CaN) or 100 μg mL^-1^ ampicillin only (CaMKII). After reaching an OD600 of 0.8–1.0, protein expression was induced with 1 mM IPTG. Concurrently, 500 μM myristic acid (Myr) was added to cultures expressing calcineurin (CaN) and 500 μM 12-azidododecanoic acid (12-ADA) was added to cultures expressing the engineered CaM. Ca^2+^/calmodulin dependent kinase II (CaMKII) cultures were not supplemented with Myr or 12-ADA. After 3–4 hours of protein expression, final OD600 readings were recorded and cultures were centrifuged to harvest cells (12,000 x g, 15 minutes, 4°C). Cell pellets were washed with cold PBS and stored at -80°C until use. Cell lysis was achieved via sonication (Branson 450 Sonifier) and proteins were subsequently collected from clarified lysate supernatents.

### Calmodulin purification

Phenyl Sepharose 6 Fast Flow (Sigma-Aldrich, St. Louis, MO) was used to purify the 12-ADA CaM by hydrophobic interaction chromatography similar to Gaertner *et al*., [16] taking advantage of the fact that Ca^2+^ binding to CaM exposes hydrophobic regions involved in many binding dynamics [4, 16, 17] that is readily reversed with Ca^2+^ chelation. Briefly, 250 mL of *E*. *coli* culture containing overexpressed 12-ADA CaM were pelleted, washed in cold PBS, resuspended in 5 mL g^-1^ lysis buffer (50 mM Tris [pH 7.5], 100 mM KCl, 1 mM EGTA, 1 mM EDTA, 1 mM DTT, 0.1 mM PMSF), and lysed via sonication. Clarified lysate was obtained by centrifugation at 12,500 x g for 20 minutes. Clarified lysate was applied to Phenyl Sepharose 6 Fast Flow resin (8 mL) equilibrated with lysis buffer. Flow through containing 12-ADA CaM was collected, supplemented with 5 mM CaCl_2_ and applied to another column (8 mL) of Phenyl Sepharose 6 Fast Flow resin pre-equilibrated with binding buffer (50 mM Tris [pH 7.5], 3 mM CaCl_2_, 0.1 mM PMSF). After washing with 5 column volumes of Wash Buffer 1 (50 mM Tris [pH 7.5], 1 mM CaCl_2_), 5 column volumes of Wash Buffer 2 (50 mM Tris [pH 7.5], 1 mM CaCl_2_, 500 mM NaCl), and 5 column volumes of Wash Buffer 1, 12-ADA CaM was eluted from the column with Elution Buffer (50 mM Tris [pH 7.5], 2 mM EGTA) resulting in high concentrations of pure 12-ADA CaM (Figure A in S1 Appendix). The protein concentration of elution fractions was quantified using Pierce 660 Protein Assay and purity was evaluated with SDS-PAGE (4–20% Mini-PROTEAN®TGX™ Precast Gels, Bio-Rad, Hercules, CA). Elution fractions containing the highest concentrations of CaM were then pooled and dialyzed in 20 mM HEPES (pH 7.5) and stored at -80°C until use.

### Covalent attachment of 12-ADA CaM to Sepharose resins

12-ADA CaM affinity resins were prepared according to previous publication [4]. In summary, one column volume of 12.5 mM dibenzocyclooctyne-(polyethylene glycol)_4_-amine (DBCO-PEG_4_-amine, Fig 1) (Click Chemistry Tools, Scottsdale, AZ) in 50% v/v DMSO and 20 mM HEPES (pH 7.5) was added to N-hydroxysuccinimide (NHS) activated Sepharose 4 Fast Flow (NHS-Sepharose, GE Healthcare Life Sciences, Marlborough, MA) to generate a strain-promoted azide-alkyne cycloaddition (SPAAC) resin (DBCO-Sepharose) (reaction shown schematically in Figure B in S1 Appendix). To generate copper-catalyzed azide-alkyne cycloaddition (CuAAC) resins (reaction shown schematically in Figure B in S1 Appendix)., one column volume of 12.5 mM, 25 mM, or 50 mM alkyne-(polyethylene glycol)_4_-amine (alkyne-PEG_4_-amine, Fig 1) (Click Chemistry Tools, Scottsdale, AZ) was added to NHS-Sepharose (GE Healthcare Life Sciences). DBCO-PEG_4_-amine and alkyne-PEG_4_-amine solutions were incubated with the NHS-Sepharose on a rotator for 2–3 hours at room temperature. Free NHS-esters were inactivated with 1 M Tris (pH 8.0) for 1 hour. The DBCO-Sepharose resin was then incubated with 1 column volume of either purified 12-ADA CaM (in 20 mM HEPES, pH 7.5) or 12-ADA CaM in clarified lysate at indicated concentrations overnight at 4°C on a rotator. The alkyne-Sepharose resins were incubated with 1 column volume of purified 12-ADA CaM in the presence of 2 mM CuSO_4_, 10 mM THPTA, and 10 mM CH_7_NaO_6_, similar to Hong et al [18]. Post-conjugation, the resins were pelleted using centrifugation (800 x g, 2 minutes) and the supernatant was removed for analysis. The 12-ADA CaM conjugated resins were then washed sequentially with 10 column volumes of the following wash buffers: Wash 1 (100 mM ammonium carbonate [pH 8.6], 2 mM EGTA), Wash 2 (10 mM Tris [pH 7.5], 1 M NaCl, 2 mM CaCl_2_), and Wash 3 (100 mM sodium acetate [pH 4.6], 2 mM CaCl_2_) and stored in 20 mM Tris (pH 7.5) with 20% EtOH prior to use.

### Determination of CaM coupling efficiency

12-ADA CaM concentration pre- and post- incubation with functionalized Sepharose was measured with Pierce 660 Protein Assay. Concentration values were subtracted from each other, resulting in the quantity (mg mL^-1^) of successful 12-ADA CaM binding to each functionalized click chemistry group on the resin.

### Purification and quantification of CaM binding partners

Calcineurin (CaN) and calcium-calmodulin-dependent protein kinase (CaMKII) purification with CaM affinity resin was performed largely according to previously published methods, with minor adjustments [15, 19]. Functionalized 12-ADA CaM resins were equilibrated in lysis buffer, depending on the target protein of interest; CaN lysis buffer consisted of 25 mM Tris (pH 7.5), 3 mM MgCl_2_, 100 mM NaCl, 2 mM CaCl_2_, lysozyme (1mg mL^-1^), Roche cOmplete™ Protease Inhibitor Cocktail tablets, and 0.1 mM PMSF, and CaMKII Lysis buffer consisted of 40 mM Tris (pH 7.5), 0.5 mM EGTA, 200 mM NaCl, 1 mM DTT, lysozyme (1mg mL^-1^), Roche cOmplete™ Protease Inhibitor Cocktail tablets, and 0.1 mM PMSF. Clarified lysates (4 mL) were added to resin (4 mL) pre-equilibrated in respective lysis buffers and incubated on a rotator at 4°C for 45 minutes -1 hr. Following protein binding, resins were pelleted with centrifugation (800 x g for 2 minutes) and unbound protein was removed. The resins were then washed sequentially with 3 column volumes of: wash buffer A (25 mM Tris, 2 mM CaCl_2_, 0.1 mM EDTA, 0.5 mM DTT, pH 7.4), wash buffer B (25 mM Tris, 2 mM CaCl_2_, 0.1 mM EDTA, 1 M NaCl, 0.5 mM DTT pH 7.4) and wash buffer A once more. CaN was eluted with 4 column volumes of buffer C (25 mM Tris [pH 7.5], 3 mM MgSO_4_, 1 mM EGTA, 0.5 mM DTT). CaMKII was eluted with 8 column volumes of buffer D (40 mM Tris [pH 7.5], 1 mM DTT, 200 mM NaCl, 2 mM EGTA). Fractions were analyzed for purified protein with SDS-PAGE (Bio-Rad 4–20% Mini-PROTEAN®TGX™ Precast Gels) and Pierce 600 Protein Assay. Semi-quantitative Coomassie gels were used to determine the amount of pure protein obtained in elution fractions using known concentrations of BSA (66 kDa) and Lysozyme (14 kDa) as standards. This allowed for quantification of the purified protein only as one-step purification rarely results in 100% pure protein. Software on the Odyssey CLx program (LI-COR) was used to determine integrated pixel intensities for standards and CaN α (60 kDa) and CaN β (19 kDa), which were then converted to protein concentration (mg mL^-1^). Data files relevant to the quantification of pure CaN may be accessed at figshare under “Coomassie-stained gels used in semi-quantitative analysis of purified calcineurin from calmodulin Sepharose resins.” https://doi.org/10.6084/m9.figshare.6270545.v1 and are available from the Purdue University Research Repository at URL: https://purr.purdue.edu/publications/2954/1 DOI:10.4231/R7Q81B9G.

### Western blot analysis of purified calcineurin

Elution fractions containing CaN were collected as described above in ‘*Purification and quantification of CaM binding partners*’. In preparation for SDS-PAGE analysis, Laemmli buffer containing 5% 2-mercaptoethanol was added to each protein-containing elution fraction at a 1:3 ratio. Fractions were boiled at 95°C for 5 minutes and were loaded equally by volume into a 4–15% gradient gel (PROTEAN®TGX™ Precast Gels, Bio-Rad). Proteins were transferred to a 0.2 μm PVDF membrane (ThermoFisher Scientific, St. Louis, MO) and probed with primary (Abcam ab52761 for α-subunit and ab49658 for β-subunit) and secondary antibodies (IRDye® 800CW and 680RD, LI-COR), then analyzed with Odyssey CLx imaging system (LI-COR).

### Calmodulin peptide quantification

CaM conjugated resins were trypsinized to obtain information about the quantity of CaM bound to the bead surfaces using Pierce™ MS-grade trypsin protease (ThermoFisher Scientific, St. Louis, MO). GE CaM Sepharose and our 12-ADA conjugated resins were equilibrated in 100 mM Tris (pH 7.5) and 2 mM CaCl_2_ to result in a 50% slurry. Next, 1 μg trypsin was added to the mixture and incubated at 37°C overnight. Samples were centrifuged (1000 x g, 5 minutes) to terminate peptide cleavage, and supernatant was removed for analysis. Peptide quantification was executed according to the Pierce™ Quantitative Colorimetric Peptide Assay (ThermoFisher Scientific, St. Louis, MO) protocol.

## Results and discussion

### Resins prepared with lower concentrations of purified 12-ADA CaM outperform those with higher concentrations

**Fig 2:**
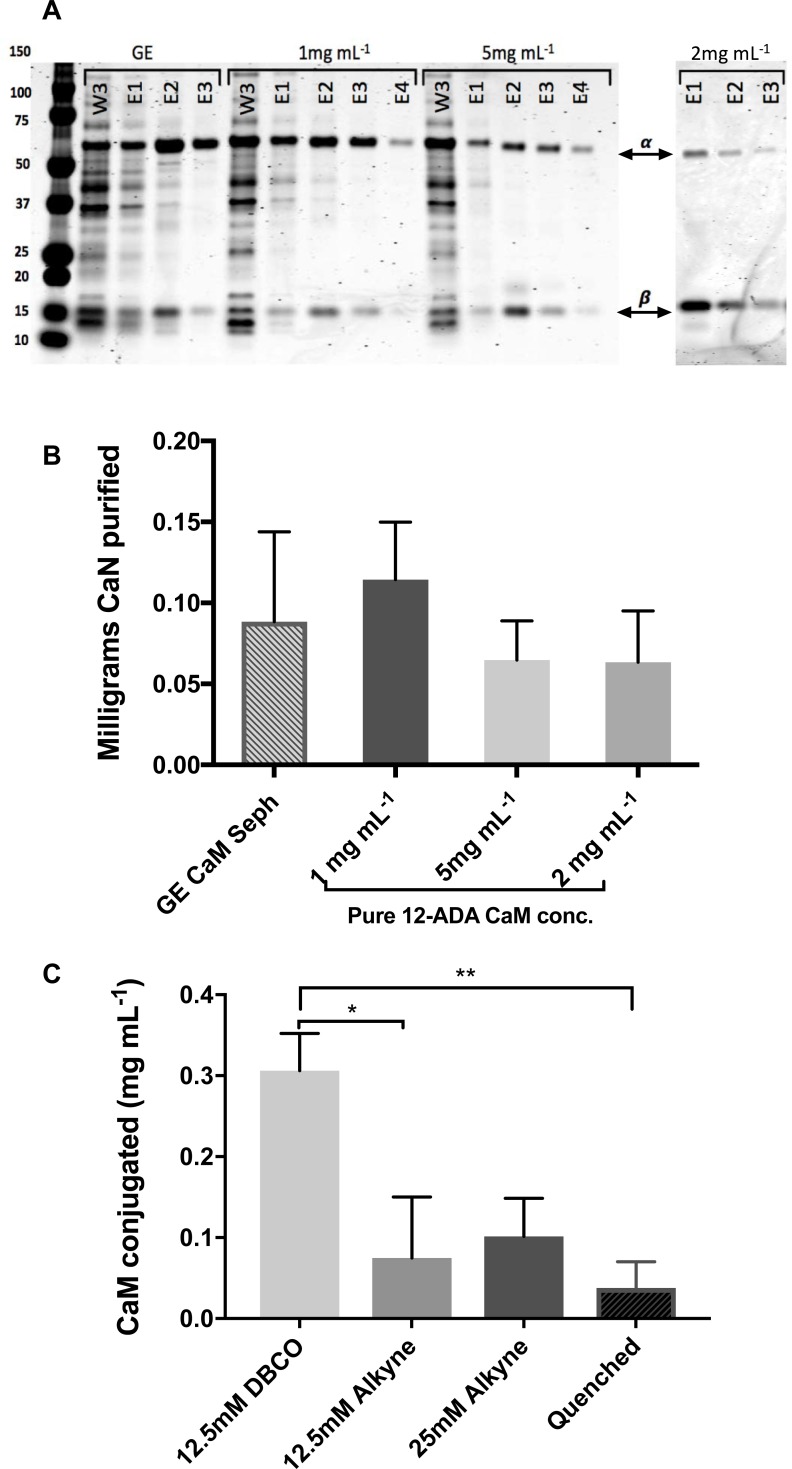
Calcineurin (CaN) purification with various CaM resins. (A) Representative SDS-PAGE gels with Coomassie stain from CaN purifications done in parallel. Calmodulin Sephaorse 4B from GE (GE) is compared to 12-ADA CaM resins made with 1, 2, and 5 mg mL^-1^ purified 12-ADA CaM. Both the α and β subunits of CaN appear at the correct MW (60 and 19 kDa, respectively). (B) Average total mg of CaN purified for each resin (n≥3). Repeats of Calmodulin Sepharose 4B were from different lots. Repeats of each resin made with different concentrations of 12-ADA CaM were made with different lots of DBCO-PEG_4_-amine and NHS-Sepharose. Higher quantities of CaN indicate better resin performance and thus reflect ideal CaM concentration. GE = commercial standard Calmodulin Sepharose 4B, 1mg mL^-1^ = resin prepared with 1 mg mL^-1^ pure 12-ADA CaM, 5 mg mL^-1^ = resin prepared with 5 mg mL^-1^ pure 12-ADA CaM, Lysate = resin prepared with 12-ADA CaM straight from crude cell lysate, W = wash, E = elution. (C) Comparison of resins made with click chemistry functional groups at different concentrations (n≥3). Total average amount of 12-ADA CaM bound to the resins as calculated by measuring the concentration of 12-ADA CaM in solution before and after conjugation. DBCO is resin prepared with DBCO-PEG_4_-amine. Alkyne are resins prepared with alkyne-PEG_4_-amine. Quenched is resin that was incubated with 1M Tris to deactivate NHS-esters instead of a click chemistry functional group. * = p≤0.05, ** = p≤0.01.

In order to maximize resin yield of purified CaM-binding protein, we first sought to optimize the amount of 12-ADA CaM that was covalently bound to the resin. Purified 12-ADA CaM at varying concentrations was incubated with dibenzocyclooctyne (DBCO) functionalized Sepharose resin to generate a series of 12-ADA CaM affinity resins. To assess the ability of each resin to purify a CaM-binding partner, calcineurin (CaN) was purified as described in the Methods. The amount of CaN (mg) purified from the 12-ADA CaM resins and the commercially available Calmodulin Sepharose 4B resin (GE Healthcare Life Sciences, Marlborough, MA) in parallel experiments was assessed by semi-quantitative SDS-PAGE (Fig 2A and 2B, Table A in S1 Appendix). Despite our prediction that resins exposed to higher concentrations of 12-ADA CaM would result in higher yields of purified CaN, we found that resins prepared with pure 12-ADA CaM at a concentration of 1 mg mL^-1^ regularly outperformed those prepared with either 2 mg mL^-1^ or 5 mg mL^-1^ (Fig 2B, Table A in S1 Appendix). The results also show that the resins made with 1 mg mL^-1^ 12-ADA CaM outperforms the commonly used Calmodulin Sepharose 4B in purifying CaN in terms of overall yield (Fig 2B, Table A in S1 Appendix).

### Optimization of CaM conjugation to resin based on click chemistry functionality

Due to its size and hydrophobic properties, the DBCO-PEG_4_-amine (Click Chemistry Tools) has limited solubility beyond 12.5 mM in 50% v/v DMSO and 20 mM HEPES (pH 7.5). We hypothesized that another click chemistry functional group, alkyne-PEG_4_-amine (Click Chemistry Tools), could produce a higher performing resin. Alkyne-PEG_4_-amine is a smaller and less hydrophobic molecule than DBCO-PEG_4_-amine, which allowed us to prepare higher concentrations of alkyne for activation of the resin, such as 25 mM and 50 mM in 50% v/v DMSO and 20 mM HEPES (pH 7.5). We anticipated that a higher concentration would result in more alkyne groups available on the resin for conjugating 12-ADA CaM, thereby increasing purification performance. Another significant difference between the two click chemistry functional groups (DBCO vs alkyne) is the buffer conditions of the azide-alkyne cycloaddition reaction. The reaction between 12-ADA CaM and DBCO reaction is strain-promoted (no need for catalyst, ligand or reducing agents) [20]. The reaction between 12-ADA CaM and alkyne is copper-catalyzed and has more stringent requirements in the buffer conditions [18].

Various concentrations of DBCO-PEG_4_-amine and alkyne-PEG_4_-amine were incubated with prepared NHS-Sepharose as described in Methods and were subsequently conjugated with 1 mg mL^-1^ purified 12-ADA CaM. The amount of 12-ADA CaM that was conjugated to the resin was estimated by measuring the amount of 12-ADA CaM in solution before and after exposure to the resin. We observed that resins prepared with 12.5 mM DBCO-PEG_4_-amine showed a significantly higher concentration of 12-ADA CaM conjugated to the resin compared to alkyne-PEG_4_-amine resins and the negative control group (Fig 2C). From these findings, we are ideally poised to move forward with DBCO as the functional group with which to efficiently conjugate 12-ADA CaM to the resin.

### Novel affinity resin prepared with 12-ADA CaM from cell lysate

**Fig 3:**
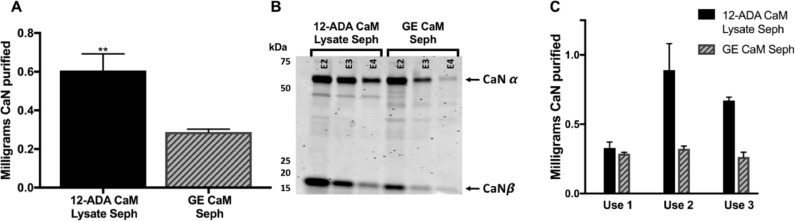
Comparison of Sepharose resins. (A) Comparison between resin made with 12-ADA CaM from clarified lysate (12-ADA CaM Lysate Seph) and Calmodulin Sepharose 4B (GE CaM Seph) (n = 12) ** = p≤0.01. (B) Representative SDS-PAGE with Coomassie staining of elution fractions containing highest amount of purified calcineurin for GE CaM Seph and resin made with 12-ADA CaM from clarified lysate. CaN α and β subunits are marked and appear at the correct MW (60 kDa and 19 kDa, respectively). (C) Results indicating the capacity of the resins to purify CaN with repeated use. n = 4 for each use.

One advantage of using azide-alkyne cycloadditon to conjugate 12-ADA CaM to the resin is that the reaction is bioorthogonal and can be performed in complex matrices like cell lysate. 12-ADA CaM was conjugated to the DBCO-functionalized resin directly from clarified cell lysate containing overexpressed 12-ADA CaM without prior purification. We compared the ability of this 12-ADA CaM-affinity resin to purify CaN to that of the widely used Calmodulin Sepharose 4B (GE Healthcare Life Sciences). In parallel experiments resins made with 12-ADA CaM from lysate consistently purified more CaN than the commercially available CaM Sepharose resin from GE. (Fig 3A and 3B).

Interestingly, resin that was made by conjugating 12-ADA CaM in clarified lysate to DBCO-PEG_4_-amine was more efficient in purifying CaN than those made from pure 12-ADA CaM (Table 1). Indeed, more than a five-fold improvement is observed between 12-ADA CaM from clarified lysate and pure 12-ADA CaM (1mg mL^-1^). One possible explanation for this result is that 12-ADA CaM from clarified lysate is conjugated to the resins on the same day it is extracted from *E*. *coli* cells, making it a one-step process. The pure 12-ADA CaM, however, is exposed to conditions in the purification process and storage at -80°C that could negatively impact CaM’s effectiveness as a binding partner and result in less overall purified CaN. Comparing the amount of CaN purified from Calmodulin Sepharose 4B (n = 15) to resin made from 12-ADA CaM in lysate (n = 12) indicates that resin made with 12-ADA CaM from clarified lysate consistently purified more CaN (Table 1). Given that this affinity purification resin is readily made without having to purify 12-ADA CaM prior to conjugation to the Sepharose, and the improved performance relative to the commercially available CaM affinity purification resin, we have termed the resin made with 12-ADA CaM from clarified lysate “next generation CaM affinity resin”.

**Table 1:** Average amount of CaN purified from CaM resins.

CaM affinity resin	Click Chemistry Functionality	Avg. amount (mg) CaN purified (n)
**12-ADA CaM Lysate**	DBCO-PEG4-amine	0.6 ± 0.03 (n)
**12-ADA Pure (1 mg mL^-1^)**	DBCO-PEG4-amine	0.1 ± 0.08 (n)
**GE CaM Sepharose**	-	0.2 ± 0.05 (n)

The average amount of CaN purified from Sepharose resins prepared with 12-ADA CaM from clarified lysates (12-ADA CaM Lysate), purified 12-ADA CaM (12-ADA Pure), and commercial Calmodulin Sepharose 4B (GE CaM Sepharose).

To characterize the ability of our resin to be regenerated and used for multiple purifications, we collected data after one, two, and three uses (Fig 3). The results indicate that the performance (amount of CaN purified) of CaM Sepharose 4B remains consistent over time. Interestingly, the performance by our next generation CaM affinity resin upon first use is consistent with the CaM Sepharose 4B resin, but improves upon subsequent uses. Multiple resin preparation washes were performed in order to improve the performance of the first use of the next generation CaM affinity resin (high NaCl, low pH, high pH, with CaCl_2_, and with excess EGTA) but the trend observed in Fig 3C remained. Wash conditions with high NaCl, low pH, and high pH were expected to disrupt electrostatic interactions, while adding excess EGTA was expected to disrupt hydrophobic interactions similar to our purification of CaM (see Methods). Regardless of wash conditions, the performance of the next generation CaM affinity resin improved upon second use. In addition, we also observed that CaN purified using next generation CaM affinity resin was significantly more pure than CaN purified using GE’s CaM Sephaorse 4B (Figure C in S1 Appendix). This is consistent with the increased amount of CaN that was purified from the next generation CaM affinity resin and indicates that 12-ADA CaM conjugated to the resin is highly active.

### Calmodulin peptide quantification and comparison between next generation CaM affinity resin and Calmodulin Sepharose 4B

We have observed that our next generation CaM affinity resin purifies more protein per milliliter resin than commercial resin (Fig 3, Table 1). This observation is likely be attributed to the mechanism of CaM immobilization. Utilizing the N-terminal azide on 12-ADA CaM for conjugation ensures site-specific binding of 12-ADA CaM to the DBCO-functionalized Sepharose resin. The commercial resin is likely made using the traditional method of CaM Sepharose affinity resin generation that takes advantage of primary amine conjugation via cyanogen bromide activated Sepharose [21, 22]. This likely results in heterogeneous presentation of CaM on resin surfaces. Thus, the differences that we see in resin performance could be due to two different factors stemming from the conjugation: first, more 12-ADA CaM may be conjugated per milliliter resin, or second, the conjugated 12-ADA CaM is more active due to the site-specific 12-ADA tag and homogeneous presentation on the surface relative to the primary amine coupling of the commercial resin.

**Fig 4:**
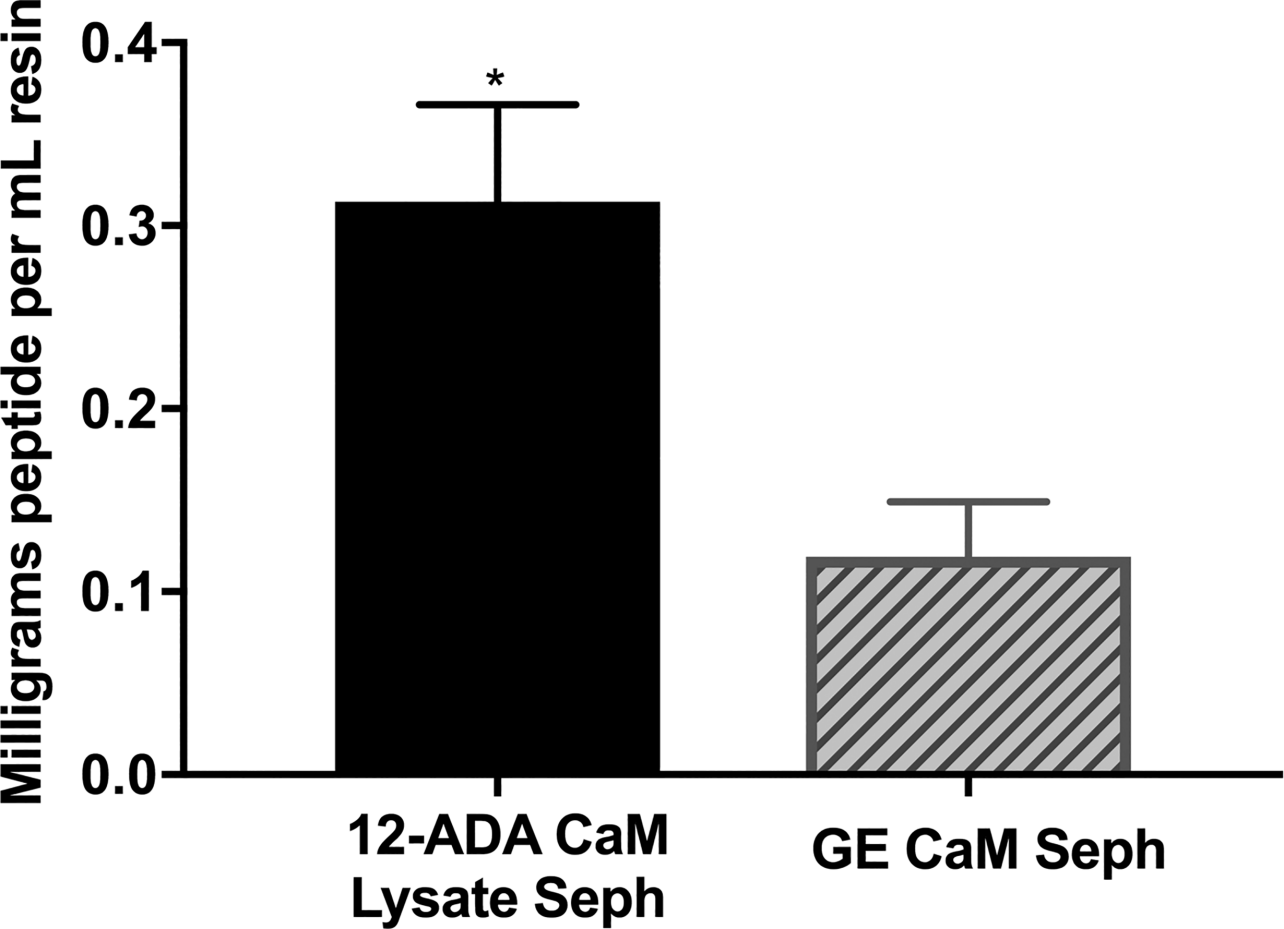
Quantification of peptides cleaved from CaM affinity resins. Trypsin digestion of the resins followed by colormetric quantification indicates that more peptide was cleaved from the next generation CaM affinity resin generated by conjugation of 12-ADA CaM from clarified lysate than from the commercially available CaM affinity resin (GE CaM Seph). * = p≤0.05 n = 3.

We sought to determine the amount of CaM on both resins by cleaving CaM peptides off our next generation affinity resin and Calmodulin Sepharose 4B via trypsin digestion. Of note, other colorimetric and absorbance-based protein quantification methods were tried, but significant background readings from the Sepharose resin interfered with those more direct quantification methods. As seen in Fig 4, significantly more peptide was obtained from our next generation affinity resin. These results indicate that the next generation CaM affinity resin had higher amounts of CaM bound per mL of resin. While these results do not distinguish between the specific activity of conjugated 12-ADA CaM on the next generation affinity resin versus CaM on the commercial resin, they do indicate that conjugation of 12-ADA to the resin directly from lysate is a highly efficient and effective conjugation method that produces an affinity resin with a maximal amount of CaM immobilized on the resin.

### Purification of a second binding partner: Ca^2+^/calmodulin-dependent kinase II

We explored the ability of the next generation CaM affinity resin to purify a second CaM-binding partner, validating the range and success of our resin. Calcium/calmodulin dependent kinase II alpha (CaMKII), a serine/threonine-specific protein kinase, is regulated by the Ca^2+^/CaM complex and is involved in numerous signaling cascades [23, 24]. Three-dimensional reconstruction has shown the full dodecameric assembly of CaMKII when isolated is around 650 kDa, however when split into individual subunits the molecular weight approximately 50 kDa [25]. CaMKII is a difficult protein to isolate with high purity and in large quantities and therefore served as a challenging target protein with which to test our resin’s robustness [19]. Traditional purification methods to isolate and enrich CaMKII include immobilized metal ion affinity chromatography (IMAC), ion exchange chromatography (typically with DEAE), as well as CaM affinity resins. Most recent CaMKII structural work has taken advantage of IMAC purification in conjugation with hexahistadine tagged CaMKII [26–28].

**Fig 5:**
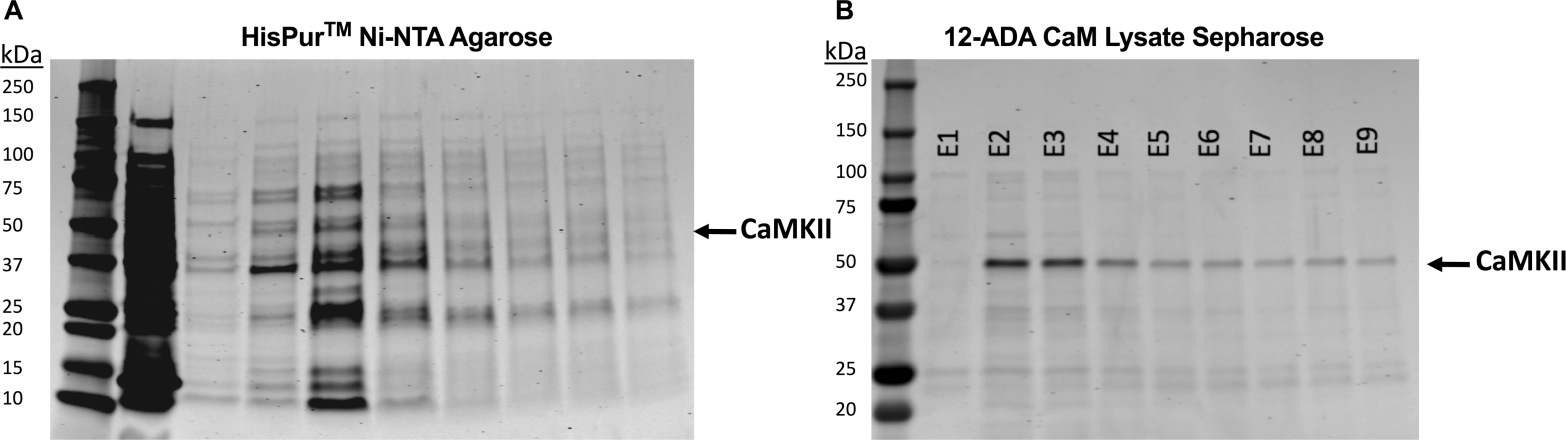
CaMKII purification. Representative SDS-PAGE of Ca^2+^/calmodulin-dependent kinase II (CaMKII) purified with two different types of affinity resins, comparing both quantity and quality of pure result. CaMKII appears at 50 kDa and is of most interest in this figure. A) Tradition methods using His-tagged affinity Ni-NTA resin B) our affinity resin generated by functionalizing NHS Sepharose with 12-ADA labeled CaM from cell lysate. CL = clarified lysate, E = elution.

In the current work C-terminally hexahistadine tagged CaMKII was recombinantly expressed in *E*. *coli* and purified in parallel experiments with our next generation CaM affinity resin and a Ni-NTA resin. Elution fractions were analyzed via SDS-PAGE, allowing both purity and quantity of CaMKII purified by two different mechanisms to be analyzed (Fig 5). It has been shown that in *E*. *coli* expression systems the C-terminus of CaMKII is truncated [29]. Degradation products appear at 37 kDa and 25 kDa, while the full length CaMKII appears as a band at 50 kDa. Elution fractions from Ni-NTA purification were considerably less pure than those of our next generation CaM affinity resin after a one-step purification (compare Fig 5A to Fig 5B). Significant amounts of degradation products were seen in elution fractions of Ni-NTA affinity resin (Fig 5A), while the majority of the elution fractions from our next generation CaM affinity resin was the 50 kDa full-length CaMKII protein (Fig 5B). Approximately 0.1 mg/mL CaMKII was obtained from 2 mL of next generation CaM affinity resin (Table B in S1 Appendix). Together, these results indicate that our resin could be used to purify a variety of CaM-binding partners with consistency and improved efficiency as compared with current methods.

## Conclusion

**Fig 6:**
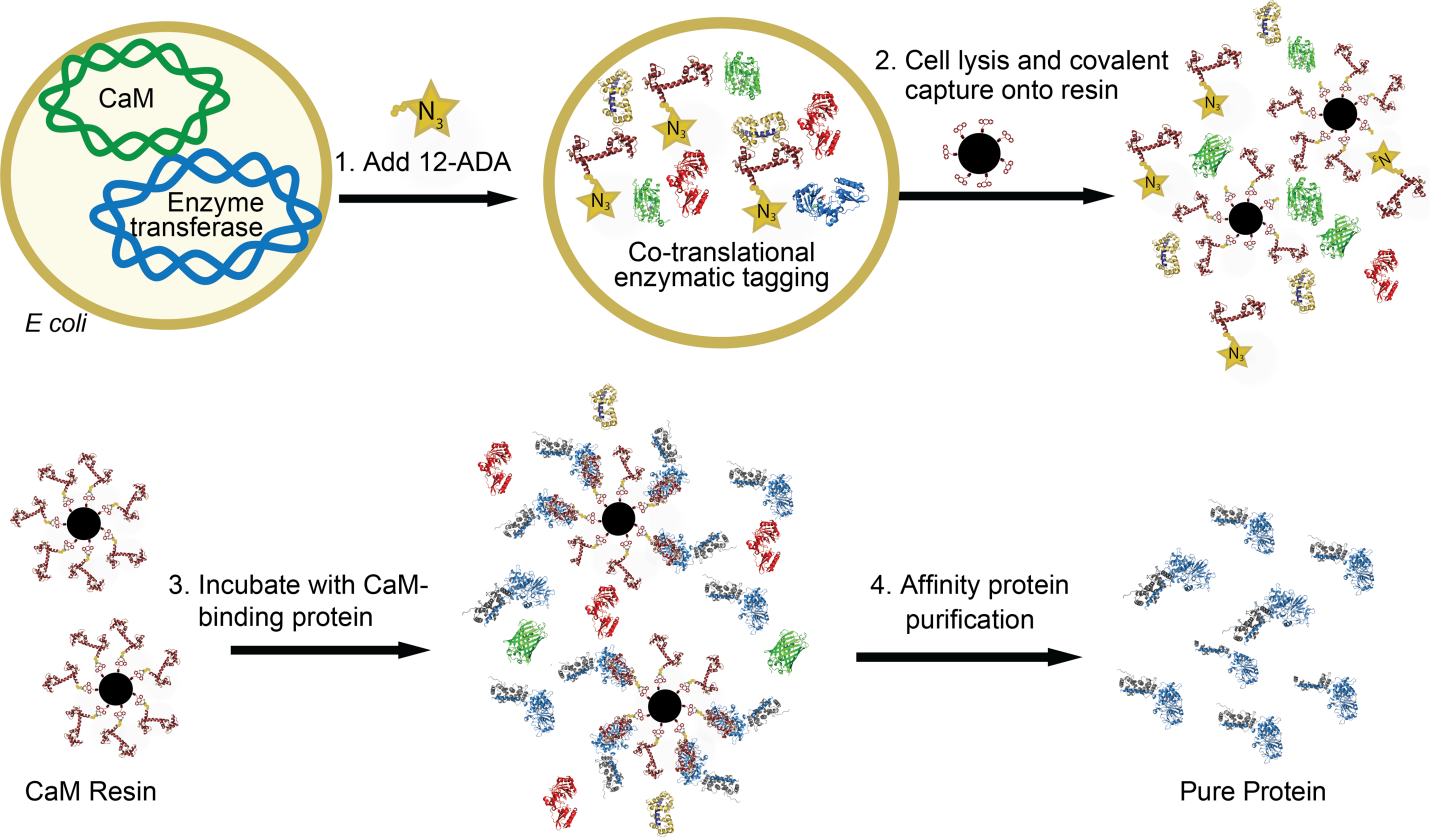
Production and use of next generation CaM resin. Overview of the production of our next generation CaM affinity resin and CaM-binding protein purification. Schematic shows the entire process sequentially. 1. Recombinant protein expression and co-translational labeling of engineered CaM with 12-ADA (terminal azide group denoted with N_3_ stars). 2. Incubation of clarified lysate containing the 12-ADA CaM with DBCO-functionalized Sepharose resin (black spheres) where 12-ADA CaM covalently conjugates to the resin. 3. Washed next generation CaM affinity resin is incubated with a crude mixture containing the CaM-binding protein to be purified. 4. After washing and elution from the resin, purified CaM-binding protein is obtained.

The generation of our next generation CaM affinity resin consists of two primary steps as depicted schematically in Fig 6: first, CaM is engineered to carry an N-myristoyl transferase recognition peptide at its N-terminus and is co-expressed in *E*. *coli* with N-myristoyl transferase where CaM alone is selectively and co-translationally modified with 12-ADA [4, 11]. Then, the cells are lysed, clarified and incubated with DBCO-functionalized Sepharose resin where the 12-ADA CaM is covalently conjugated to the resin via the SPAAC reaction (Fig 6 top). The result is a fully functional next generation CaM affinity resin that can be used to purify a variety of CaM binding proteins to high purity in a one-step purification process (Fig 6 bottom).

The main advantage of using 12-ADA CaM to generate these resins is that CaM is highly expressed and co-translationally tagged with 12-ADA in *E*. *coli* and 12-ADA CaM is readily covalently conjugated to resin via click chemistry directly from cell lysate without having to purify the 12-ADA CaM. We have also shown that this produces a CaM affinity resin that outperforms commercially available CaM affinity resin in terms of both purity and quantity of purified calcineurin, and outperforms IMAC affinity chromotrogaphy in purifying CaMKII. We expect that other CaM-binding proteins would also be successfully purified using our next generation CaM affinity resin.

We expect that affinity chromatography resins that conjugate other proteins beside CaM to the resin could also be generated in this way. N-terminal myristoylation-enabled tagging with 12-ADA is highly selective in recombinant *E*. *coli* systems [11]. The click chemistry functionality of 12-ADA and other similar myristic acid analogs allows for bioorthogonal conjugation of the N-terminally labeled protein of interest directly from cell lysate [4, 30, 31], which significantly streamlines the affinity resin production process.

## Supporting information

S1 Appendix Includes supplemental figures and tables showing an overview of click chemistry and data for quantitative analysis. (DOCX)
