# Supplementary material for: Next generation calmodulin affinity purification: Clickable calmodulin facilitates improved protein purification
Source: PLoS One. 2018 Jun 4;13(6):e0197120. doi: 10.1371/journal.pone.0197120 (PMC5986150; doi:10.1371/journal.pone.0197120)
Supplement: S1 Appendix — (DOCX) [file pone.0197120.s001.docx]

**S1 Appendix**

**Contents:**

Figure A. Purification of 12-ADA CaM

Figure B. Click chemistry reactions

Table A. CaM concentrations used in experiments

Figure C. Purity analysis comparing GE CaM Seph and 12-ADA CaM Lysate Seph

Table B. Quantification of pure CaMKII


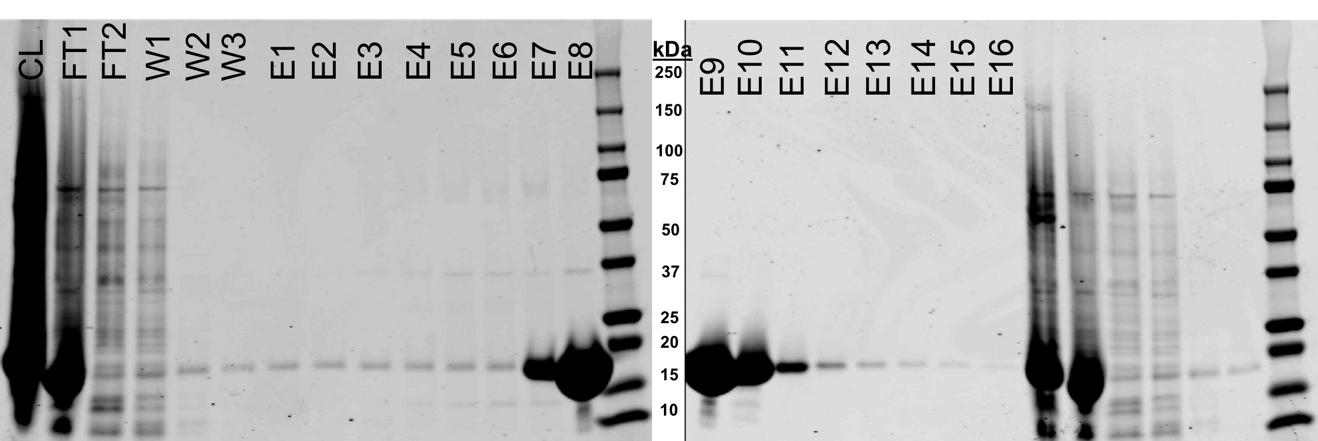


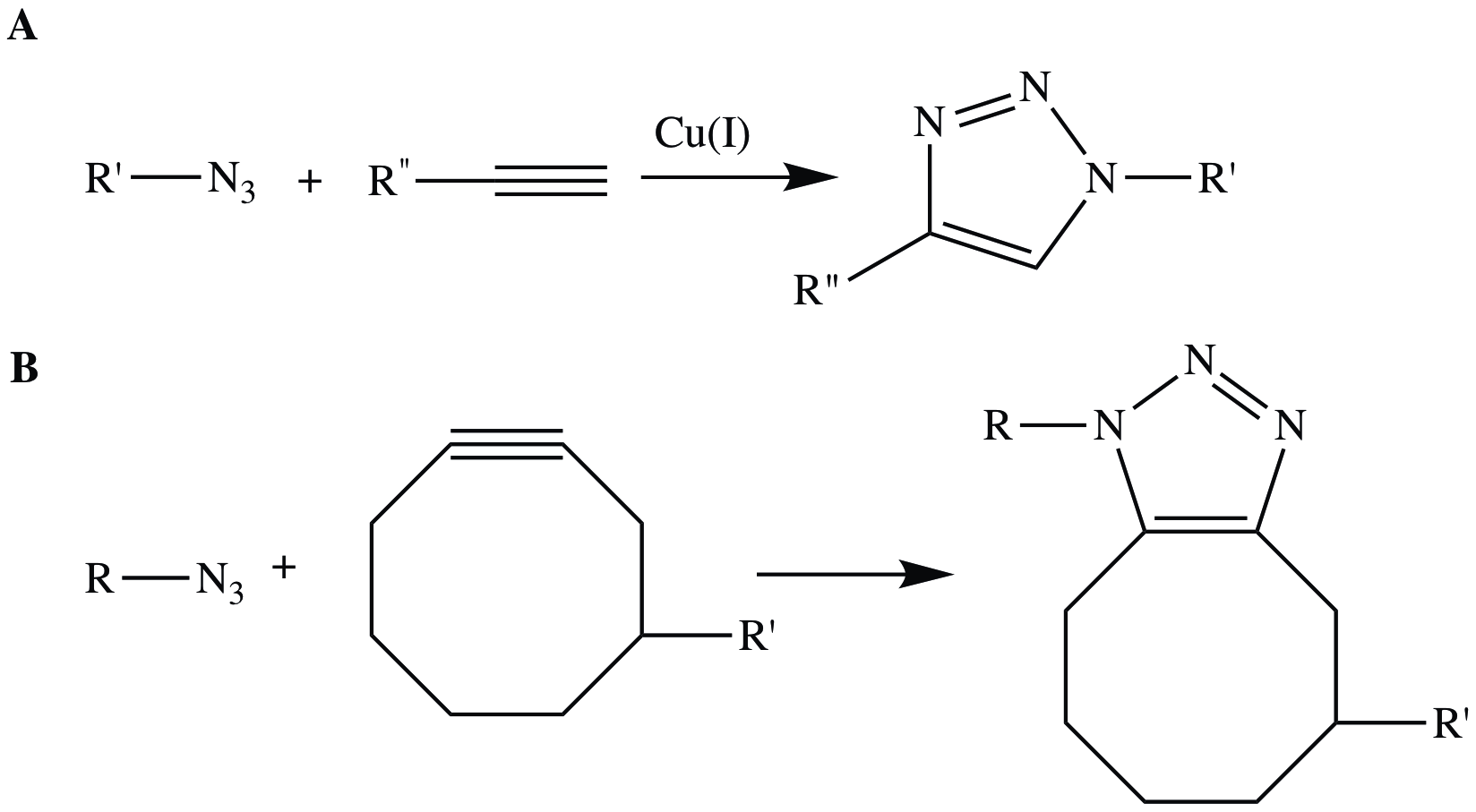


| **Protein Solution** | **Total protein concentration (mg mL^-1^)** | **CaM concentration (mg mL^-1^)** | **CaM concentration (mM)** |
| --- | --- | --- | --- |
| Purified 12-ADA CaM | - | 1 | 59 |
| Purified 12-ADA CaM | - | 2 | 118 |
| Purified 12-ADA CaM | - | 5 | 294 |
| Clarified Lysate with overexpressed 12-ADA CaM (15%)* | 3.5 | 0.5 | 31 |
| Clarified Lysate with overexpressed 12-ADA CaM (20%)* | 3.5 | 0.7 | 41 |
| *CaM was previously estimated to be 15-20% of total protein in clarified lysates[1]. | | | |


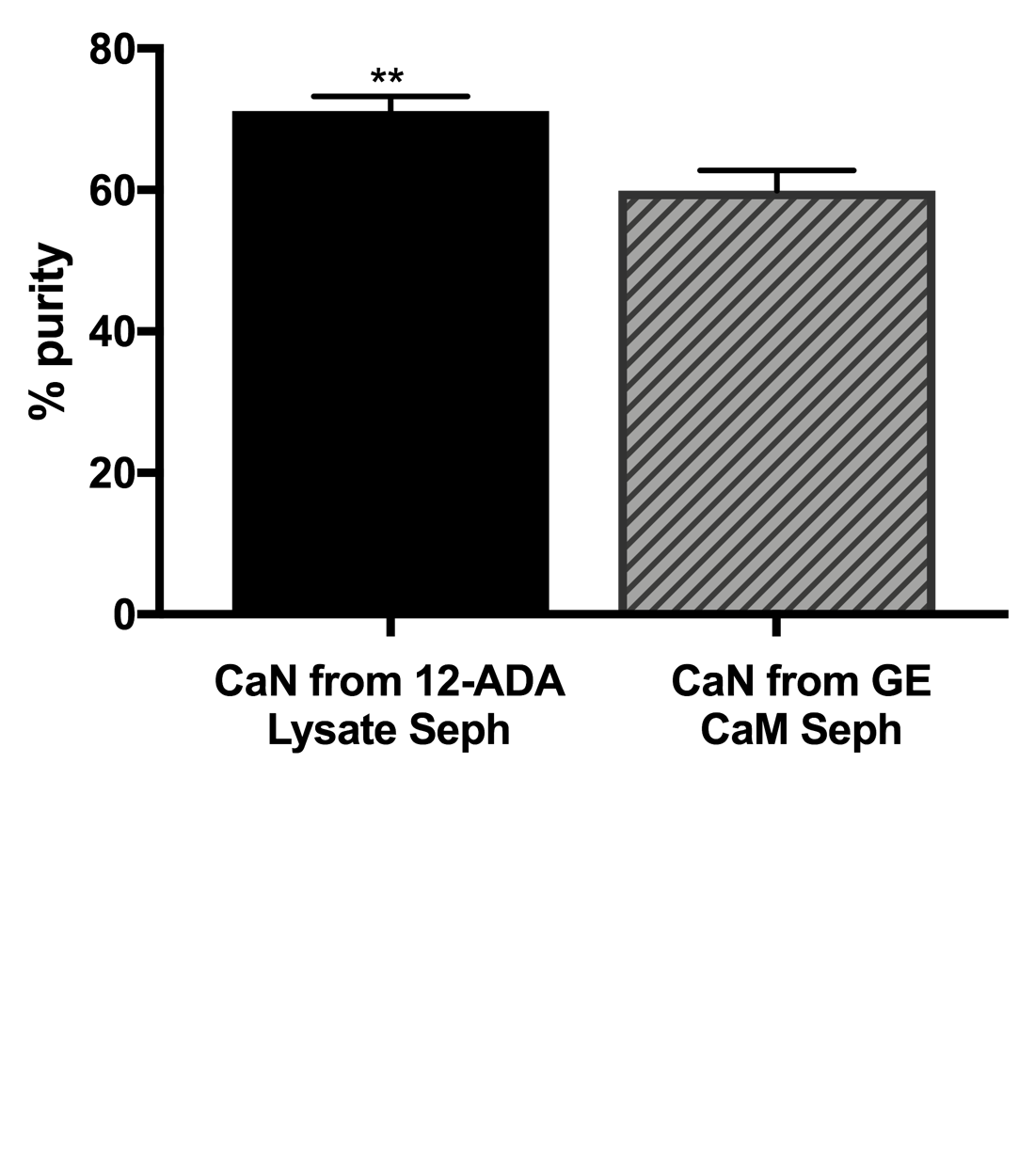


| **Elution Peaks** | **Average elution peak conc. (mg/mL)** | **Average amount purified (mg)** | **Total pure protein (mg)** |
| --- | --- | --- | --- |
| E2 | 0.02 ± 0.002 | 0.04 ± 0.004 | 0.1 |
| E3 | 0.02 ± 0.003 | 0.04 ± 0.0008 |  |
| E4 | 0.01 ± 0.01 | 0.02 ± 0.03 |  |
